# The impact of a COVID-19 lockdown on work productivity under good and poor compliance

**DOI:** 10.1093/eurpub/ckab138

**Published:** 2021-08-06

**Authors:** Julius Ohrnberger, Alexa Blair Segal, Giovanni Forchini, Marisa Miraldo, Janetta Skarp, Gemma Nedjati-Gilani, Daniel J Laydon, Azra Ghani, Neil M Ferguson, Katharina Hauck

**Affiliations:** 1 School of Public Health, MRC Centre for Global Infectious Disease Analysis, Imperial College London, London, UK; 2 Department of Management & Centre for Health Economics & Policy Innovation, Imperial College London, London, UK; 3 Department of Economics, Umeå School of Business, Umeå University, Umeå, Sweden

## Abstract

**Background:**

In response to the COVID-19 pandemic, governments across the globe have imposed strict social distancing measures. Public compliance to such measures is essential for their success, yet the economic consequences of compliance are unknown. This is the first study to analyze the effects of good compliance compared with poor compliance to a COVID-19 suppression strategy (i.e. lockdown) on work productivity.

**Methods:**

We estimate the differences in work productivity comparing a scenario of good compliance with one of poor compliance to the UK government COVID-19 suppression strategy. We use projections of the impact of the UK suppression strategy on mortality and morbidity from an individual-based epidemiological model combined with an economic model representative of the labour force in Wales and England.

**Results:**

We find that productivity effects of good compliance significantly exceed those of poor compliance and increase with the duration of the lockdown. After 3 months of the lockdown, work productivity in good compliance is £398.58 million higher compared with that of poor compliance; 75% of the differences is explained by productivity effects due to morbidity and non-health reasons and 25% attributed to avoided losses due to pre-mature mortality.

**Conclusion:**

Good compliance to social distancing measures exceeds positive economic effects, in addition to health benefits. This is an important finding for current economic and health policy. It highlights the importance to set clear guidelines for the public, to build trust and support for the rules and if necessary, to enforce good compliance to social distancing measures.

## Introduction

Since the December 2019 outbreak in China, the novel coronavirus virus (COVID-19) has become a global pandemic. The short-term health impact of the pandemic is unprecedented, with nearly 470 000 deaths globally, and about 45 000 of these deaths coming from the UK.^1^ In response to control the spread of the pandemic and to minimize both mortality and the strain on NHS hospital capacities, on 23 March 2020, the UK government implemented a ‘lockdown’. Lasting until the beginning of June 2020, this period included the non-pharmaceutical interventions (NPIs) case isolation, social distancing of the entire population, household quarantine and business, school and university closure.^2^^,^^3^ Public compliance with these measures is necessary for the effective suppression of COVID-19, as lockdown ensures that the spread of COVID-19 is lowered. Whilst the health benefits of good public compliance vs. poor compliance to these NPIs in response to pandemic outbreaks have been clearly identified, the economic effects are unknown.^4–6^

Compliance behaviour to laws, orders and public rules can be motivated by demographic, instrumental or normative factors.^7^ Demographic factors such as gender or age can predict compliance with research finding that men and younger individuals are less compliant than women and older individuals.^8^ Normative factors relate to people’s perceived duty to support the authorities and/or to act for the greater good of the society.^9^^,^^10^ Instrumental factors are motivated by individual returns of compliant behaviour by weighing the benefits of an action against the costs of the action.^11^^,^^12^ Whilst systematic reviews show that various studies have analyzed the determinants and health effects of compliance to lockdown measures of pandemics that have occurred in the 20th and 21st century,^13^^,^^14^ none has to our knowledge focused on identifying the effects of compliance to containment measures on work productivity, and in doing so to quantify this instrumental societal value of compliance.

Therefore, in this article, we add to the literature by estimating and comparing the work productivity effects of good public compliance to poor public compliance to the UK lockdown measures among the employed Welsh and English labour force. To our knowledge, this research is the first to identity productivity effects of compliance to COVID-19-related lockdown policies. To measure the impact of COVID-19 on mortality and morbidity, we combine a labour force model with outputs from the epidemiological individual-based spatial model by Ferguson et al. (2020) who simulate the effect of good and poor compliance of the UK lockdown policy on the spread of COVID-19.^6^^,^^15^

Our study also contributes beyond COVID-19. Findings from our analysis contribute to the debate of the impacts of individual vs. social preferences in health and work contexts. One can understand good compliance being in line with societal preferences and poor compliance closer aligning to individual preferences, circumstances and socio-economic factors, including the ability to work from home (WFH). It can be individually rational to defect NPIs due to economic necessity, or preferences in socializing or work environments, especially for low-risk populations. Often individuals can or do not consider the wider societal consequences of their actions for other people’s health and the related economic consequences.^16^ By comparing the work productivity effects of a good and a poor compliance scenario, we also illustrate the economic effects of such behaviours.

Furthermore, the findings of our analyses provide inputs for economic analyses, such as cost-benefit analyses of lockdown policies or macro-economic studies estimating the financial burden of the pandemic.^17^ Beyond the empirical contributions, our study also adds to methodological advances by developing a framework that integrates epidemiological predictions into an economic micro-simulation model. An advantage of the framework is that it can be modified to be used in the analysis of NPI effects on work productivity of other infectious diseases in different contexts.

## Methods

### Suppression strategy

We build our analysis on projections of COVID-19 spread in the population of England and Wales coming from the individual-based simulation model (IBM) by Ferguson et al. (2020). Prior studies have used this IBM to provide predictions of the incidence of infection and mortality rates by infection group.^4–6^ Ferguson et al. (2020) have modelled the suppression strategy (widely referred to as lockdown) and tested the effect on the transition dynamics of COVID-19.

The suppression measures implemented in the lockdown scenario include case isolation, social distancing of the entire population, household quarantine, reducing workplace contacts and school and university closure.^6^ In this article, the good compliance scenario assumes social distancing leads to a 90% drop in social contacts and 75% compliance with household quarantine, while poor compliance assumes social distancing leads to a 66% drop in social contacts and 50% compliance with household quarantine. Both scenarios assume only 50% of workplaces remain open during lockdown, but the good compliance scenario assumes 50% effective social distancing within open workplaces while the poor compliance scenario assumes social distancing within open workplaces only reduces infectious contacts by 25%. A reproduction number, *R*_0_, of 2.8 was assumed for both compliance scenarios. The model provides results as four main outputs—the daily population prevalence of (i) uninfected individuals, (ii) infected but not hospitalized individuals, (iii) hospitalized cases and (iv) cumulative deaths in England and Wales.

We simulate the effects on productivity, applying weekly averages of the IBM simulation outputs, over the period of 13 weeks which is the time, from 16 March until 7 June 2020, covering the time of the UK-lock down. Since compliance levels vary across different population groups and compliance levels impact productivity differentially, a core aspect of our analyses is to consider different compliance scenarios. It is important to note that we do not model mixed-compliance behaviours. As such, we estimate ceiling effects using two scenarios of good and poor compliance. Simulation analysis is here appropriate as two hypothetical scenarios are compared.^18^ The reader will find a brief discussion on the value of simulation analyses in the health economic and public health context in the appendix.

### Productivity effects

The different productivity effects of the lockdown on the UK labour force vary by worker groups, defined as keyworkers and all other non-essential workers. To identify channels of work productivity effects, we use the government job roles and classification of COVID-19 lockdown-specific keyworkers and key-industries/businesses^19^ and information of sectoral options to WFH^20^ to characterize the labour force into four groups:

- Group I: keyworkers in key sectors such as healthcare, food and beverages sector (including production, distribution and retail), transport and utilities. Individuals in this group may still use child-care services and schools irrespective of closures of schools or nurseries following government guidelines^19^;
- Group II: non-essential workers whose occupation and nature of work tasks do not allow working from home (e.g. individuals working in the manufacturing industry or wholesale and retail businesses allowed to remain open under the isolation policy)^20^^,^^21^;
- Group III: non-essential workers in businesses that had to close temporarily due to government policy (e.g. individuals working in restaurants, hospitality industry and retailers)^19^;
- Group IV: non-essential workers whose occupation allows working from home (e.g. workers from tertiary education, certain financial businesses or human resource services).^20^^,^^21^

We provide a detailed list of the composition of the four groups in Table A3 in the supplemental material. We discuss in the following section how productivity effects of the four groups vary between the good and poor compliance scenario.

### Channels of impact

COVID-19 affects productivity in three ways, with heterogenous impacts by policies but also through different channels:

1. *Sickness*, where the effect of sickness varies for hospitalized and mild cases. Hospitalized workers do not work and therefore experience a full productivity loss over the period of the hospital stay.^22^ We assume that cases with mild illness in the group of home office workers are either absent from work or work despite feeling sick with is reflected in a loss of productivity of about 60% (*absenteeism & presenteeism*). Cases in groups I and II who cannot WFH are assumed to be absent from work over the period of their mild sickness.^23^
2. *Premature mortality* resulting in a loss of individual productivity from the age at death until retirement.
3. *Non-health-related reasons* which cause productivity losses as a result of a decreased ability to WFH or increased household responsibilities such as caring for children due to school and nursery closure or from business closures following the UK lockdown policies.

### Productivity effects due to morbidity and non-health-related reasons

We measure productivity effects from morbidity (sickness) and non-health-related effects with the average weekly gross income in each of the defined four groups. Using the average weekly income as measure for productivity is a standard approach and follows the assumption that marginal productivity is equal to the marginal cost.^24–26^

Table 1 provides an overview of the different types of productivity effects for the four labour force groups. Group I, the keyworkers, are affected by losses in their productivity due to sickness and hospitalization. We assume that no further losses occur as keyworkers may still send their children to school or day care.^19^

**Table 1:** Types of work productivity effects by labour force group

Productivity effect	Group I: keyworkers (workplace or home)	Group II: workers non-essential (workplace)	Group III: workers non-essential (workplace closed)	Group IV: workers non-essential (home)
Mildly sick (absenteeism)	×	×	Full loss of productivity	
Mildly sick and working from home (presenteeism)				×
Hospitalization	×	×		×
Working from home (WFH)^a^				×
WFH single parent				×
WFH full parenting support^a^				×
WFH shared parenting support				×
Single parent		×		
Full parenting support		×		
Shared parenting support		×		

a Affects only ‘WFH’ and ‘WFH full parenting support’ subgroups as these groups are not constrained in their choice of the workplace location by child caring duties.

For workers in Group II, non-essential workers without the possibility to WFH, there are two sources of productivity losses: the losses related to their own illness and mortality, as well as productivity losses arising from childcare duties due to school and nursery closures for single parents (*Single parent*) or parents living with only employed household members with whom child caring duties are assumed to be shared (*Shared parenting support*). We assume zero productivity losses where parents can rely on an unemployed adult household member to care for the child(ren) (*Full parenting support*). We assume zero productivity in Group III due to government enforced shut down of businesses for employed workers who cannot WFH.

For Group IV, non-essential workers that can WFH, we measure lost productivity arising from two channels: sickness and presenteeism resulting from other competing tasks in the household such as childcare. Non-health-related productivity losses for this group are caused by decreased ability to perform work-related tasks due to several potential factors: being a single parent with no other household member to share childcare duties (*WFH single parent*), living with an employed partner or other employed household member where child caring duties are assumed to be shared (*WFH shared parenting support*) or living with an unemployed partner or adult household member who can look after the child (*WFH full parenting support)*. These subgroups are assumed to have no child-care obligations and can work either from home or their workplace.

### Productivity effects due to premature mortality

With regards to productivity effects due to premature mortality, we compute productivity losses due to premature mortality using the human capital approach, computing the net present value (NPV) of overall lost life-income as a result of premature death.^24^^,^^26^ Following the methodology, the NPV of lost income is defined by the lost life-years until retirement at age 65, multiplied by discounted annual income which is used to compute the NPV. Assuming that mortality is invariant between Groups I, II, III and IV, we compute mortality effects over the whole employed labour force by 10-year age-groups (16–24, 25–34, 35–44, 45–54, 55–64). We thereby combine mortality estimates from the IBM for the 10-year age-groups and the average annual income in each age-group. We compute the NPV of forgone productivity with a discount factor drawn from a uniform distribution between 3 and 5% to assess sensitivity, shown in Table A1 of the appendix.^27–29^

### Labour force model—data

We used the Office for National Statistics Quarterly Labour Force Survey (QLFS) for data from 1 October through 31 December 2019, to build a static labour force baseline model (pre-COVID-19). QLFS is the largest representative household survey of the UK population, including approximately 40 000 households and 80 000 individuals per quarter.^30^ Households are randomly selected by postcode within a geographical area and followed up for five waves (five quarters) and then replaced with a new cohort. QLFS provides detailed data about individual employment conditions including employment status and occupation, data on household composition including age of household members, household structure and the number of dependent children living in the household. QLFS comes with calibrated income and population weights which we use in this analysis, as suggested by the Office for National Statistics (ONS).^30^

We focus on the salaried economically productive population and constrain our analytical QLFS sample accordingly. The sample is composed of individuals who are of working age (16–64 years), report to be employed and are English and Welsh residents, restricting the QLFS to 33 128 individuals. The sample is restricted to England and Wales because the IBM model was calibrated on these populations. Using population weights in the analysis, our sample is representative of the Welsh and English active labour force of about 28 million people. We acknowledge that unemployed and retired individuals can also have productivity effects on the economy; however, due to data limitations, we cannot assess effects on either of these population groups. Previous studies analysing the economic impacts of pandemics also excluded the unemployed and retired population.^26^^,^^31–34^

Using the QLFS, we compute indicators of the percentage of individuals living without children (*WFH*), with children as single parents (*WFH single parent; Single parent*), living with children and an adult in the household who is unemployed and can look after the child(ren) (*WFH full parenting support*; *Full parenting support*) or who is employed and with whom child caring duties are shared (*WFH shared parenting support*; *Shared parenting support*) (see table 1). We use information on individual occupation (ICDM codes) to group individuals into the I–IV Groups and compute the total weekly wage income for each of the I–IV Groups at baseline (pre-COVID-19). We then use QLFS age information to compute 10-year age bands and the total annual wage income in each age-band.

Productivity effects related to morbidity and non-health reasons are identified by multiplying IBM average weekly predictions of population proportions of (i) non-infected and asymptomatic infected; (ii) mildly sick not requiring hospitalization; (iii) infected requiring hospitalization (IBM predictions) with the total weekly wage income in each of the four groups (Group III is always zero per assumption); and (iv) proportions of family composition, productivity for keyworkers and non-essential workers and home office efficacy parameters, where applicable by groups (QLFS). A detailed description and the parameterization of the model is provided in the supplemental material.

## Results

**Figure 1:**
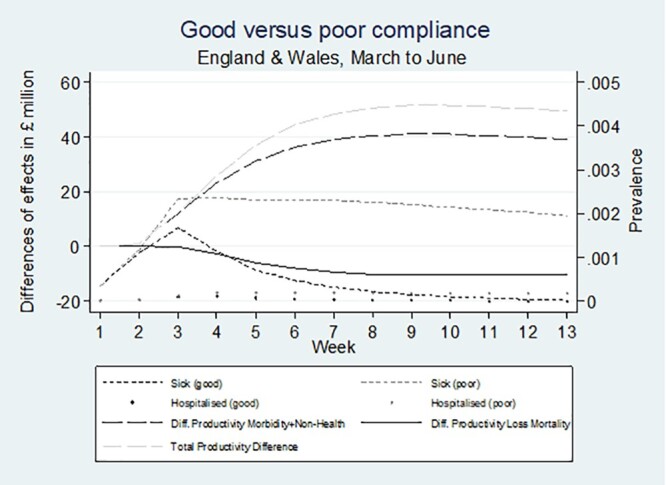
Differences in mortality losses, work productivity and total work productivity: good and poor compliance

Figure 1 illustrates the evolution of differences in work productivity between the good and poor compliance scenarios by morbidity and non-health reasons (black long dashed line), mortality (black line) and total aggregated differences (light grey long dashed line) over the 13-week period of the UK lock down. We compute the figure using mean values of parameters. The differences of productivity effects are expressed in £million with higher values in morbidity and non-health reasons and total aggregated differences indicating higher productivity in good compliance. The NPV of losses in lifetime productivity due to premature mortality are expressed in £million, with higher values indicating more losses due to increased mortality.

Included in figure 1 is the population prevalence of hospitalized and non-hospitalized sick cases for each compliance scenario. The short dashed grey line presents the prevalence of sick people in the poor compliance scenario. Prevalence of sick cases among the working population peaks after around 3 weeks at 0.0025% and smoothly reduces to 0.002% until week 13 at the end of the lockdown. In comparison, prevalence of sick people in the good compliance scenario, expressed by the short dashed black line, peaks at a same time at < 0.002% but approaches 0% at about the end of the lockdown. Hospitalized cases in both scenarios (grey dots for poor compliance and black dots for good compliance) follow similar comparative trends, but at much lower prevalence levels and lagging the sickness curves.

Values for mortality losses are negative from week 3 onwards implying higher mortality losses in the poor compliance scenario. Differences in productivity effects due to morbidity or non-health reasons are positive meaning that work productivity is always higher in the good compliance scenario. Productivity differences increase from week 2 until week 9 where they reach a maximum difference of about £40 million. Differences marginally decrease from week 9 onwards due to reduced sickness prevalence in poor compliance. The total differences in productivity which accounts for both mortality losses and productivity losses follows a similar pattern to the productivity difference, reaching its peak at week 9 at £50 million and decreasing from thereon slightly.

In table 2, we present the aggregated productivity effects of good compliance vs. poor compliance to lockdown rules, where, as described in the previous section: (i) productivity effects due to morbidity and non-health reasons, (ii) losses in productivity due to premature mortality and (iii) the total productivity effects. We split the analyses by the duration of the lockdown, ranging from 1 month until 3 months, the end of the lockdown. The effects are estimated in £million.

**Table 2:** Work productivity effects of good compliance vs. poor compliance

Duration of lockdown	Differences of productivity effects	Mean effect (£ million)	Standard error	95% Confidence interval
1 month (weeks 1–4): March–April	1: Morbidity + non-health (Good–Poor)	29.21	0.07	(29.08–29.34)
	2: Mortality loss (Good–Poor)	–2.70	0.00	(–2.70–2.69)
	3: Total productivity effect (1 – 2)	31.91	0.07	(31.78–32.04)
2 months (weeks 1–8): March–May	1: Morbidity + non-health (Good–Poor)	147.32	0.33	(146.67–147.96)
	2: Mortality loss (Good–Poor)	−36.24	0.04	(–36.32 to –36.16)
	3: Total productivity effect (1–2)	183.56	0.33	(182.90–184.21)
3 months (weeks 1–13): March–June	1: Morbidity + non-health (Good–Poor)	310.05	0.70	(308.68–311.42)
	2: Mortality loss (Good–Poor)	−88.53	0.10	(–88.73 to –88.33)
	3: Total productivity effect (1–2)	398.58	0.71	(397.19–399.97)

Note: 95% confidence intervals in (brackets). Monte Carlo simulation with 1000 repetitions. Positive values in 1 imply higher work productivity driven by health and non-heath reasons due to good compliance compared with poor compliance; Negative values in 2 imply reduced losses in life-long productivity due to mortality comparing good compliance to poor compliance; positive values in 3 imply total productivity gains in good compliance compared with poor compliance.

One month into the lockdown, good compliance is significantly associated with a £31.91 million gain in total productivity, with £29.21 million in productivity gains due morbidity and non-health reasons, and an averted loss of £2.7 million in mortality. These total productivity gains of good compliance increase after 3 months, the end of the national UK lockdown, to £398.58 million. This is due to falling sickness prevalence in good compliance and averted mortalities. Avoided losses mortality are £88 million. Productivity gains in good compliance due to morbidity and non-health reasons are £310 million.

Table 3 presents the total aggregated differences in productivity of good and poor compliance per individual worker expressed in £. We find that on average good compliance improves work productivity per worker by £1.34 after 1 month into lockdown, this increases to 7.72 after 2 months of lockdown and to £16.76 at the end of the lockdown. The non-linear increases of productivity gains over time are explained by the non-linear nature of the epidemiology of COVID-19 effects on sickness prevalence and mortality.

**Table 3:** Differences in work productivity effects per worker in good compliance vs. poor compliance due to health and non-health reasons

Duration of lockdown	Mean effect per worker (£)	Standard error	95% confidence interval
1 month (weeks 1–4): March–April	1.34	0.003	(1.34; 1.35)
2 months (weeks 1–8): March–May	7.72	0.01	(7.69; 7.74)
3 months (weeks 1–13): March–June	16.76	0.03	(16.71–16.82)

Note: 95% confidence intervals in (brackets). Monte Carlo simulation with 1000 repetitions. Values in £ per worker. Positive values imply total productivity gains in good compliance compared with poor compliance.

We test the sensitivity of our findings with regards to the timing of returning to work after hospitalization. Doing so, we add an extra week of full absenteeism from work for those individuals with a more severe case of COVID-19 but did not die because of the disease. The findings are presented in Table A2 in the supplemental material and corroborate our findings in table 2.

## Discussion

Our results provide evidence that good compliance (compared with poor compliance) to lockdown regulations shows strong positive economic effects in the short-term, at £31.91 million after 1 month, and £398.58 million after 3 months, the end of the UK lockdown. Differences are driven by the effect that good compliance has in halting the spread of the disease and flattened the epidemic curve and thusly, decreases in morbidity and premature mortality losses whilst accounting for non-health-related reasons. About 75% of the work productivity effects are due to differences in non-health and morbidity effects and 25% are explained by premature mortality effects between the two scenarios. The lower share of avoided mortality losses can be explained by the demographic composition of the labour force, as younger age groups are less at risk of COVID-19-related mortality.^6^ The differences in morbidity and non-health reasons can be interpreted as avoided cost of absenteeism in good compliance.

Whilst our analysis has used the features of the first UK lockdown alongside specific COVID-19 population predictions, the implications of our findings are valid in similar contexts such as Western European countries. These countries apply similar lockdown policies while also sharing comparable social welfare systems that implement furlough schemes, technological standards permitting working from home, comparable health care systems and demographic structures which affect both work productivity effects and the transmission of COVID-19.

Our analysis has some limitations. We may underestimate productivity effects as healthy individuals that are staying at home can produce other goods and services (e.g. childcare, informal care, cleaning, cooking). This includes the retired, the unemployed, as well as those not in the labour force. Due to data limitations, we cannot infer productivity measures for non-market activities and consequentially output and mortality effects for these populations. However, our approach is consistent with the literature which uses measures such as GDP—which neither accounts for nonmarket productivity nor mortality effects of the non-working populations.^31–35^

We use a static labour force model without accounting for labour force dynamics of policy scenarios, such as job-shifting patterns. We thus estimate a ceiling effect in productivity of good vs. poor compliance. However, we account for reductions in overall economic productivity by allowing uncertainty in sectoral productivity. We do not model the effect of intra-household COVID-19 effects, e.g. sickness of the partner or quarantine effects arising due partner’s ill health and related consequences for productivity. Both limitations may result in partly underestimating the productivity losses due to morbidity comparing the good compliance scenario to the poor compliance scenario.

Our study does not consider the potential negative effects of compliance to social distancing on social isolation, lack of belonging or relatedness. Previous research showed that increased loneliness or isolation can reduce economic outcomes.^36^ Such effects are difficult to quantify in monetary terms, and we have therefore not included them in our analysis. Future research should evaluate the role of such factors in work productivity.

Our analysis relies on various assumptions of parameter values. All parameters and their distributions are informed by existing surveys and studies. Moreover, we randomly draw from parameter distributions and hence incorporate uncertainty about the ‘true’ values of the parameters and variations of parameters between individuals. We are therefore confident that neither the parameters nor their values lack generalizability, or specific parameter values drive the results.

Our simulation makes a unique contribution to research by combining daily epidemiological IBM predictions that, different to basic Susceptible-Infected-Recovered (SIR)-models, account for individual heterogeneity, with a labour-economic micro-simulation producing more realistic simulations of the studied population over time. This a different approach to existing economic studies which rely on more basic SIR-compartmental epidemiological models and fewer data points. We are thus able to show the variation of the economic costs alongside the pandemic trajectory over time, accounting for heterogeneities in the population resulting in more realistic projections.

Our analysis corroborates findings of other works that have investigated compliance behaviour as a result of a pandemic, in that lockdown behaviours are necessary to halt the spread of disease.^13^ Gupta et al. (2005) reported that quarantine in Canada following the SARS outbreak was costly upfront, but ultimately saved lives and money for the public (saving around $200 million). A main difference between our work and that of others is that we model productivity impacts on the economy according to various lockdown periods. In doing so, we observe heterogeneity in the overall impact of total productivity by different lengths of lockdown. Our findings have important implications for policy as they highlight that good compliance has real and important effects on both health outcomes and the economy. Accordingly, it is important that governments set clear rules to the public, build trust and support for the rules and if necessary, enforce compliance.

Whilst our analysis identified the lockdown effects on work productivity in a high-income country (HIC), future research is required to assess the effects in different socio-economic settings. Such analysis should look at variations of work productivity effects among social classes within HICs, addressing the unequal impact of COVID-19 especially on lower-income groups. We would also encourage research to assess the robustness of our work in the context of low- and middle-income countries where, e.g. demographic factors strongly vary in comparison to HICs, eventually affecting both the health impacts of COVID-19 and related work productivity effects. Furthermore, future research should also explore alternative behavioural response models, such as mixed-compliance behaviour where compliance behaviour can vary within the population.

## Supplementary data

Supplementary data are available at *EURPUB* online.

## Funding

Julius Ohrnberger acknowledges financial support from the Medical Research Council Skill Development Fellowship MR/T025409/1, www.mrc.ukri.org. The funders had no role in study design, data collection and analysis, decision to publish or preparation of the manuscript.

*Conflicts of interest*: None declared.

## Data sharing agreement

The code of the individual-based epidemiological model is available via https://github.com/ImperialCollegeLondon/covid19model. The raw data from the Quarterly Labour Force Survey October to December 2019 used for the labour force model is available via https://www.ukdataservice.ac.uk/.

Key points

- We uniquely combine epidemiological prediction with economic modelling.
- We estimate COVID-19 lockdown compliance-based work productivity effects in England and Wales.
- Good compliance (compared with poor compliance) to lockdown regulations shows strong positive economic effects (£398.58 million) after 3 months, the end of the UK lockdown.
- Most of the effect (75%) is explained by morbidity and non-health reasons which can be attributed to reduced absenteeism in good compliance.
- Individual behavioural responses to the COVID-19 pandemic matter, both for containing the spread of health and the economy.

## Supplementary Material

ckab138_Supplementary_Data
