# Supplementary material for: The impact of a COVID-19 lockdown on work productivity under good and poor compliance
Source: Eur J Public Health. 2021 Aug 6;31(5):1009–15. doi: 10.1093/eurpub/ckab138 (PMC8385936; doi:10.1093/eurpub/ckab138)
Supplement: ckab138_Supplementary_Data [file ckab138_supplementary_data.docx]

**Appendix**

*A note on simulation studies*

Simulation studies are widely applied in health economic and public health research (1,2). The advantage of simulation studies or ex-ante evaluations over ex-post evaluations is that they permit the analysis of a policy or a health behaviour under different scenarios where usually such data is not available (2). In doing so, simulation studies can add important insights to the policy and research debate and inform and shape future ex-post analyses (1,2). In the context of health economic and public health research, simulation studies are often used to understand the population-wide economic costs of illness or the economic consequences of a health policy or a health behaviour (3–7). For example, Smith et al. (2011) use a macro-economic simulation to understand the economic consequences of various social distancing policies in response to a hypothetical influenza pandemic in the UK. The authors find that prophylactic work absenteeism and school closures potentially cost the UK between 1.3% and 1.4% of the GDP. This is one of the first studies to highlight that while mitigation strategies are important to control the spread of an influenza pandemic they also come at economic cost and consequences. The simulation study stimulated both policy and research, especially considering the present COVID-19 pandemic.

*Labour force model - Parameterisation*

Table A1 lists the various productivity parameters required for the simulation. For each simulation, parameters are drawn from the defined distributions. As informed by the literature, we use Monte Carlo simulation with 1000 repetitions to address uncertainty around productivity parameters and compute 95%-confidence intervals for the different estimated productivity effects (8).

Where we have information about a range of potential effects, such as working when sick with lower efficacy expressed as parameter *Presenteeism.* We draw parameters from a uniform distribution except for *Productivity Non-keyworkers,* where we use a beta-type distribution with mean value 0.8 which is informed by the literature (9–12). This assumption is required to address the wide uncertainty around the likelihood of productivity losses for non-keyworkers. It also reflects the overall reduced economic productivity affecting both the intensive and extensive margin of labour supply and hence, total labour productivity.

*Labour force model - Identifying productivity effects due to morbidity and non-health reasons*

Productivity effects related to morbidity and non-health reasons are identified by multiplying IBM average weekly predictions of population proportions of 1) non-infected and asymptomatic infected; 2) mildly sick not requiring hospitalisation; 3) infected requiring hospitalisation (IBM predictions) with the total weekly wage income in each of the four groups (Group III is always zero per assumption); and 4) proportions of family composition, productivity for keyworkers and non-essential workers and home office efficacy parameters, where applicable by groups (QLFS). Thereby, we assume that the distribution for cases 1), 2) and 3) does not vary between the four different groups. We briefly illustrate in two examples how we compute the various productivity effects outlined in table 1. Example (1): the total productivity loss due to mildly sick cases $\pi{(S)}_{\left( I \right)}$ for keyworkers (Group I) over the selected period of the simulation (1, 2, or 3 months) from the first week (16^th^ March) *t=1* until the last week of the chosen period (maximum for 3 months is 7^th^ June) *t=n* is:

$$\left( 1 \right) \pi\left( S \right)_{\left( I,i \right)}=\sum_{t=1}^{n} S_{\left( t,i \right)} (Y_{\left( I \right)}\lambda_{\left( I \right)})$$

We compute $\pi\left( S \right)_{\left( I,i \right)}$ for each scenario *i={good compliance; poor compliance*}as a summative function over the weekly varying prevalence of mildly sick $S_{\left( t,i \right)}$ times productivity $(Y_{\left( I \right)}\lambda_{\left( I \right)})$ in Group I, which is composed of the total weekly income $Y_{\left( I \right)}$ of Group I and the parameter for the productivity change $\lambda_{\left( I \right)}$ in Group I.

Example (2): the effect of mildly sick cases on productivity $\pi\left( S \right)_{\left( IV,i \right)}$ for individuals living without children in Group IV is:

$$(2) \pi\left( S \right)_{\left( IV,i \right)}=\sum_{t=1}^{n} S_{\left( t,i \right)} ( Y_{\left( IV \right)}\lambda_{\left( IV \right)}\sigma_{\left( IV \right)}\tau_{\left( IV \right)} \rho_{\left( IV \right)})$$

Different to (1), some productivity effects occur in this group per definition. Where productivity $(Y_{\left( IV \right)}\lambda_{\left( IV \right)}\sigma_{\left( IV \right)}\tau_{\left( IV \right)} \rho_{\left( IV \right)})$ is composed of $Y_{\left( IV \right)}$ the total weekly income in Group IV times$\lambda_{\left( IV \right)}$ the productivity change in Group IV times $\sigma_{\left( IV \right)}$ is the percentage of individuals without children in Group IV times $\tau_{\left( IV \right)}$ the effect of working from home on productivity times $\rho_{\left( IV \right)}$ the effect of presenteeism on productivity.

We compute productivity effects for each scenario *i* and use the poor compliance scenario counterfactual outcome to identify comparable overall productivity effects of both scenarios. To do so, we firstly compute an overall productivity $\pi_{(g,i)}$for each group *g={Group I, Group II, Group III, Group IV}* and policy scenario *i*. $\pi_{(g,i)}$is summed over productivity for healthy and asymptomatic ${\pi\left( H \right)}_{\left( g,i \right)}$ and productivity (losses) for mildly sick $\pi\left( S \right)_{\left( g,i \right)}$ and for those hospitalised $\pi\left( Hosp \right)_{\left( g,i \right)}$as described in equations (3) and (4):

$$(3) \pi_{(g,i)}= {\pi\left( H \right)}_{\left( g,i \right)} - \pi\left( S \right)_{\left( g,i \right)} - \pi\left( Hosp \right)_{\left( g,i \right)}; \mathrm{for} g=\{I,II\}$$

$$(4) \pi_{(g,i)}= {\pi\left( H \right)}_{\left( g,i \right)}+\pi\left( S \right)_{\left( g,i \right)}- \pi\left( Hosp \right)_{\left( g,i \right)}; \mathrm{for} g=IV$$

For Group I and Group II, $\pi\left( S \right)_{\left( g,i \right)}$has a negative sign as no work when sick is assumed. i.e. no presenteeism and full absenteeism. The total productivity in each scenario *i* is then the sum of all the four group outputs where the output for Group III is assumed to be zero due to the full loss of productivity.

Secondly, we compute differences in productivity effects $\pi_{\left( \Delta\right)}$ due to differences between good and poor compliance as described in equation (5):

$$\left( 5 \right)\pi_{\left( \Delta\right)}=\pi_{(G)}-\pi_{(B)}$$

Where $\pi_{\left( \Delta\right)}$ is the difference of productivity effects $\pi_{(G)}$ is the overall productivity in the good compliance scenario and $\pi_{(B)}$ is the total productivity in the poor compliance scenario. A positive $\pi_{\left( \Delta, \right)}$ indicates productivity gains of the good compliance scenario over the poor compliance scenario, whereas a negative sign indicates productivity losses.

*Labour force model - Identifying productivity losses due to premature mortality*

Productivity losses related to premature mortality are identified by multiplying IBM average weekly predictions of population incidence of mortality. We compute productivity losses due to premature mortality as follows:

$$(6) \pi(M)_{\left( a,i \right)}=\sum_{t=1}^{n} {\eta(M}_{\left( a,t,i \right)}Y_{\left( a \right)},\mu_{\left( a,t,i \right)})$$

Where $\pi(M)_{\left( a,i \right)}$is the total net present value of lost life-time productivity due to premature mortality for age group *a = {16-24; 25-34; 35-44; 45-54; 55-64}* in scenario *i={good compliance; poor compliance*} from *t=1* until *t=n* the last week of the selected analytical period. $\pi(M)_{\left( a,i \right)}$ is summed over the total weekly net present value of lost life-time productivity in age-group *a* for scenario *i*. $\eta$ is the net present value function which is informed by $M_{\left( a,t,i \right)}$ the mortality incidence for age-group *a* in week *t* for scenario *i* times $Y_{\left( a \right)}$ the total annual wage income in a and $\mu_{\left( a,t,i \right)}$ the years productive years lost until retirement due to premature mortality in age-group *a* in week *t* due to scenario *i*. We then derive the total productivity loss $\pi(M)_{\left( i \right)}$ in scenario *i* by summing over all $\pi(M)_{\left( a,i \right)}$ and take the difference $\pi(M)_{\left( G \right)}$-$\pi(M)_{\left( B \right)}$to compute differences in mortality losses between good compliance and poor compliance. Negative values indicate here reduced losses in productivity due to premature mortality in the good compliance scenario compared to the poor compliance scenario, and vice versa.

*Labour force model - Total productivity effects*

We combine premature mortality losses in productivity and morbidity and non-health related effects to compute the total difference in productivity ${\pi(total)}_{\left( \Delta\right)}$ of the good compliance scenario and the poor compliance scenario as follows:

$$(7) {\pi(total)}_{\left( \Delta\right)}=(\pi_{(G)}-\pi(M)_{\left( G \right)})-(\pi_{(B)}-\pi(M)_{\left( B \right)})$$

Where $\pi_{(G)}-\pi(M)_{\left( G \right)}$ is the full productivity effect of good compliance and $(\pi_{(B)}-\pi(M)_{\left( B \right)})$ is the full productivity effect of poor compliance. A positive value of ${\pi(total)}_{\left( \Delta\right)}$ would indicate a productivity gain of good compliance over poor compliance and a negative sign would indicate a productivity loss of good compliance compared to poor compliance.

**REFERENCES**

1. Rutter CM, Zaslavsky AM, Feuer EJ. Dynamic Microsimulation Models for Health Outcomes: A Review. Med Decis Mak [Internet]. 2010 May 18;31(1):10–8. Available from: https://doi.org/10.1177/0272989X10369005

2. Zucchelli E, Jones A, Rice N. The evaluation of health policies through dynamic microsimulation methods. Int J Microsimulation. 2012;5(1):2–20.

3. Smith RD, Keogh-Brown MR, Barnett T. Estimating the economic impact of pandemic influenza: An application of the computable general equilibrium model to the UK. Soc Sci Med [Internet]. 2011;73(2):235–44. Available from: http://dx.doi.org/10.1016/j.socscimed.2011.05.025

4. Keogh-Brown MR, Smith RD, Edmunds JW, Beutels P. The macroeconomic impact of pandemic influenza: Estimates from models of the United Kingdom, France, Belgium and the Netherlands. Eur J Heal Econ. 2010;11(6):543–54.

5. Smith RD, Keogh-Brown MR, Barnett T, Tait J. The economy-wide impact of pandemic influenza on the UK: A computable general equilibrium modelling experiment. BMJ. 2009;339(7733):1298.

6. Smith RD, Keogh-Brown MR. Macroeconomic impact of pandemic influenza and associated policies in Thailand, South Africa and Uganda. Influenza Other Respi Viruses. 2013;7(SUPPL.2):64–71.

7. Larsson S, Prioux M, Fasth T, Ternhag A, Struwe J, Dohnhammar U, et al. A microsimulation model projecting the health care costs for resistance to antibacterial drugs in Sweden. Eur J Public Health [Internet]. 2019 Jun 1;29(3):392–6. Available from: https://doi.org/10.1093/eurpub/cky209

8. Hoffman JIE. Resampling Statistics. 2015. p. 655–61.

9. McDonald JB, Ransom M. The Generalized Beta Distribution as a Model for the Distribution of Income: Estimation of Related Measures of Inequality BT - Modeling Income Distributions and Lorenz Curves. In: Chotikapanich D, editor. New York, NY: Springer New York; 2008. p. 147–66. Available from: https://doi.org/10.1007/978-0-387-72796-7_8

10. Forrest N, Hawksworth J, Kupelian B, Tuckett A, Lee E, Teow J. COVID-19 UK Economic Update, 7 April. PricewaterhouseCoopers. 2020;

11. ONS. Business Impact of COVID-19 Survey (BICS) [Internet]. 2020 [cited 2020 Apr 14]. Available from: https://www.ons.gov.uk/economy/economicoutputandproductivity/output/datasets/businessimpactofcovid19surveybics

12. ONS. Coronavirus and the economic impacts on the UK: 18 June 2020 [Internet]. 2020 [cited 2020 Jun 22]. Available from: https://www.ons.gov.uk/businessindustryandtrade/business/businessservices/bulletins/coronavirusandtheeconomicimpactsontheuk/18june2020

13. Van Wormer JJ, King JP, Gajewski A, McLean HQ, Belongia EA. Influenza and Workplace Productivity Loss in Working Adults. J Occup Environ Med. 2017;59(12):1135–9.

14. Glenn Dutcher E. The effects of telecommuting on productivity: An experimental examination. The role of dull and creative tasks. J Econ Behav Organ [Internet]. 2012;84(1):355–63. Available from: http://dx.doi.org/10.1016/j.jebo.2012.04.009

15. Sander B, Nizam A, Garrison Jr. LP, Postma MJ, Halloran ME, Longini IM. Economic evaluation of influenza pandemic mitigation strategies in the us using a stochastic microsimulation transmission model. Value Heal. 2009;12(2):226–33.

16. Milkie MA, Nomaguchi KM, Denny KE. Does the amount of time mothers spend with children or adolescents matter? J Marriage Fam. 2015;77(2):355–72.

17. Belli P, Anderson JR, Barnum HN, Dixon JA, Tan J-P. Economic Analysis of Investment Operations: analytical tools and practical applications. Washington D.C.: The International Bank for Reconstruction / The World Bank; 2001.

18. Freeman MC, Claxton C. Reviewing discount rates in ONS valuations. 2017.

19. Milne GJ, Halder N, Kelso JK. The Cost Effectiveness of Pandemic Influenza Interventions: A Pandemic Severity Based Analysis. PLoS One. 2013;8(4).

20. Office for National Statistics. Labour Force Survey User Guide - volume 1 - LFS Background and Methodology [Internet]. Vol. 1. 2018. Available from: www.impact-test.co.uk

**TABLES**

**Table A1** Parameters for productivity effects

| **Type** | **Parameter {range}/value** | **Distribution** | **Source(s)** |
| --- | --- | --- | --- |
| *Presenteeism* | {0.26, 0.41} | uniform | (13) |
| *Working from home (WFH)* | {0.9,1} | uniform | (14) |
| *WFH single parent* | {0.57,0.625} | uniform | (15,16) |
| *WFH full parenting support* | {0.9,1} | uniform | (14) |
| *WFH shared parenting support* | {0.66, 0.75} | uniform | (15,16) |
| *Discount factor* | {0.03, 0.05} | uniform | (17–19) |
| *Productivity Non-essential workers (Group II, III, IV)* | alpha:8, beta:2; mean 0.8, i.e.  20% reduction in productivity | Beta | (10,11) |
| *Productivity Keyworkers (Group I)* | {1,1.1} | uniform | (10,11) |

**Table A2 Sensitivity Analysis by timing of return to work after hospital stay: Productivity effects of good compliance versus poor compliance**

| **Duration of lockdown** | **Differences of productivity effects** | **Mean effect**  **(£ million)** | **Standard Error** | **95% Confidence Interval** |
| --- | --- | --- | --- | --- |
| **1 month**  **(weeks 1-4):**  **March-April** | 1: Morbidity + non-health (Good - Poor) | 30.00 | 0.07 | [29.86; 30.13] |
|  | 2: Mortality loss (Good - Poor) | -2.70 | 0.00 | [-2.70; -2.69] |
|  | 3: Total Productivity effect (1 - 2) | 32.69 | 0.07 | [32.56; 32.83] |
| **2 months**  **(weeks 1-8):**  **March - May** | 1: Morbidity + non-health (Good - Poor) | 149.18 | 0.33 | [148.53; 149.84] |
|  | 2: Mortality loss (Good - Poor) | -36.24 | 0.04 | [-36.32; -36.16] |
|  | 3: Total Productivity effect (1 - 2) | 185.42 | 0.34 | [184.76; 186.08] |
| **3 months**  **(weeks 1-13):**  **March-June** | 1: Morbidity + non-health (Good - Poor) | 311.88 | 0.70 | [310.51; 313.26] |
|  | 3: Mortality loss (Good - Poor) | -88.53 | 0.10 | [-88.73; -88.32] |
|  | 4: Total Productivity effect (1-2) | 400.41 | 0.71 | [399.01; 401.81] |
| *Note:* 95% Confidence Intervals in [brackets]. Monte Carlo simulation with 1000 repetitions. Positive values in 1 imply higher work productivity driven by health and non-heath reasons due to good compliance compared to poor compliance; Negative values in 2 imply reduced losses in life-long productivity due to mortality comparing good compliance to poor compliance; Positive values in 3 imply total productivity gains in good compliance compared to poor compliance. | | | | |

**Table A3** Composition of Group I to Group IV by industry class of main job

| **Group I: Key workers** | **Group II: None-key workers: no home office option** |
| --- | --- |
| 01.11 Growing of cereals, except rice | 01.43 Raising horses and other equines |
| 01.13 Growing veg & melons, roots & tuber | 02.10 Silviculture & other forestry act |
| 01.16 Growing of fibre crops | 02.20 Logging |
| 01.19 Growing of other non-perennial crop | 02.40 Support services to forestry |
| 01.21 Growing of grapes | 06.10 Extraction of crude petroleum |
| 01.25 Growing other tree, bush fruit & nut | 06.20 Extraction of natural gas |
| 01.30 Plant propagation | 07.29 Mining othr non-ferrous metal ore |
| 01.41 Raising of dairy cattle | 08.11 Quarry ornamental & building ston |
| 01.42 Raising other cattle and buffaloe | 08.12 Operation of gravel & sand pits |
| 01.45 Raising of sheep and goats | 08.91 Mining chem & fertiliser minerals |
| 01.46 Raising of swine pigs | 08.92 Extraction of peat |
| 01.47 Raising of poultry | 08.93 Extraction of salt |
| 01.49 Raising of other animals | 08.99 Other mining and quarrying n.e.c. |
| 01.50 Mixed farming | 09.10 Support actv petrol & nat gas extrac |
| 01.61 Support activities for crop production | 09.90 Supp actv other mining & quarryin |
| 01.62 Support activities for animal pro | 10.83 Processing of tea and coffee |
| 01.63 Post-harvest crop activities | 11.01 Distil, rectifyg & blending spiri |
| 01.64 Post-harvest crop activities | 11.02 Manufacture of wine from grape |
| 03.11 Marine fishing | 11.03 Manuf of cider & other fruit wine |
| 03.12 Freshwater fishing | 11.05 Manufacture of beer |
| 10.11 Processing and preserving of meat | 11.06 Manufacture of malt |
| 10.12 Proc & preserving of poultry meat | 11.07 Manu soft drinks & mineral waters |
| 10.13 Productn meat & poultry meat prod | 12.00 Manufacture of tobacco products |
| 10.20 Proc fish, crustaceans & molluscs | 13.10 Prep & spinning of textile fibres |
| 10.31 Proc and preserving of potatoes | 13.20 Weaving of textiles |
| 10.32 Manu of fruit & vegetable juice | 13.30 Finishing of textiles |
| 10.39 Other proc & presvg of fruit & ve | 13.91 Manu knitted & crocheted fabrics |
| 10.51 Operation dairies & cheese making | 13.92 Man made-up textile art, exc appl |
| 10.52 Manufacture of ice cream | 13.93 Manufacture of carpets and rugs |
| 10.61 Manufacture of grain mill product | 13.94 Man cordage, rope, twine & nettin |
| 10.62 Manu of starches & starch product | 13.95 Man non-woven & assoc art, ex app |
| 10.71 Man bread, fresh pastry gds & cak | 13.96 Manuf of other tech & ind textile |
| 10.72 Man ruskbiscpres pastry gdscake | 14.11 Manufacture of leather clothes |
| 10.81 Manufacture of sugar | 14.12 Manufacture of workwear |
| 10.82 Man cocoa, chocolate & sugar conf | 14.13 Manufacture of other outerwear |
| 10.84 Manu of condiments & seasonings | 14.14 Manufacture of underwear |
| 10.85 Manu of prepared meals & dishes | 14.19 Manu other wearing apprl & acces |
| 10.86 Man homogen food preps & diet foo | 14.20 Manufacture of articles of fur |
| 10.89 Manu other food products n.e.c. | 14.31 Manu knitted & crocheted hosiery |
| 10.91 Manu preprd feeds for farm animal | 15.11 Tanning, dressing, dye leathrfur |
| 10.92 Manufacture of prepared pet foods | 15.12 Man lug, hndbgs, sddlry & harness |
| 18.11 Printing of newspapers | 15.20 Manufacture of footwear |
| 20.15 Man fertilisers & nitro compounds | 16.10 Sawmilling and planing of wood |
| 20.20 Manu of pest & other agrochem pro | 16.21 Man ven sheets & wood-based panel |
| 20.41 Man soap & detgts clean & pol pre | 16.23 Manu of other builders |
| 21.10 Manuf of basic pharmaceutical pro | 16.24 Manufacture of wooden containers |
| 21.20 Man of pharmaceutical preparation | 16.29 Man oth prod wood & plaiting mat |
| 32.50 Man med & dental instruments & su | 17.11 Manufacture of pulp |
| 35.11 Production of electricity | 17.12 Manuf of paper and paperboard |
| 35.12 Transmission of electricity | 17.21 Man & cont corrgatd pper & pperbr |
| 35.13 Distribution of electricity | 17.22 Manu of hhold & sanittoilet goods |
| 35.14 Trade of electricity | 17.23 Manufacture of paper stationery |
| 35.21 Manufacture of gas | 17.24 Manufacture of wallpaper |
| 35.22 Dist of gaseous fuels thrgh mains | 17.29 Man othr art of ppr & pprbd n.e.c |
| 35.23 Trade of gas through mains | 18.12 Other printing |
| 36.00 Water collction, treatmnt & suppl | 18.13 Pre-press and pre-media services |
| 37.00 Sewerage | 18.14 Binding and related services |
| 38.11 Collection of non-hazardous waste | 18.20 Reproduction of recorded media |
| 38.12 Collection of hazardous waste | 19.20 Manu of refined petroleum prod |
| 38.21 Treatmnt & disp of non-hazrd wast | 20.11 Manufacture of industrial gases |
| 38.22 Treatmnt & disp of hazrdous waste | 20.12 Manufacture of dyes and pigments |
| 39.00 Remdiatn actv & oth wste mgmt ser | 20.13 Manu other inorganic basic chem |
| 46.11 Agnts inv in sale of agri raw mat | 20.14 Manuf of other organic basic chem |
| 46.17 Agnts inv in sale food, bev & tob | 20.16 Manuf of plastics in primary form |
| 46.23 Wholesale of live animals | 20.17 Manu synth rubber in primary form |
| 46.31 Wholesale of fruit and vegetables | 20.30 Manu of paints & related products |
| 46.32 Whlesale of meat and meat product | 20.42 Man perfumes & toilet preparation |
| 46.33 Wsale dairy prod, edible oilsfats | 20.52 Manufacture of glues |
| 46.36 Wsale of sugar & choc & sugar con | 20.53 Manufacture of essential oils |
| 46.37 Wsale coffee, tea, cocoa & spices | 20.59 Manu of other chemical prod n.e.c |
| 46.38 Wsale of oth food, inc seafood | 20.60 Manufacture of man-made fibres |
| 46.46 Wholesale of pharmaceutical goods | 22.11 Manu, retread of rub tyres & tube |
| 47.11 Ret sale non-spec str foodbevtob | 22.19 Manuf of other rubber products |
| 47.21 Ret sale fruit & veg in spec stor | 22.21 Man plastic plates, sheets, tubes |
| 47.22 Ret sale meat & rel prod spec str | 22.22 Manuf of plastic packing goods |
| 47.23 Ret sale of seafood in spec store | 22.23 Manuf of builders? ware of plasti |
| 47.24 Ret sale of bakery prod spec stre | 22.29 Manuf of other plastic products |
| 47.29 Othr ret sale of food in spec str | 23.11 Manufacture of flat glass |
| 47.73 Disp chemist in specsd stores | 23.12 Shaping and procesng of flat glas |
| 47.74 Ret sale of med eqmt in spec stre | 23.13 Manufacture of hollow glass |
| 47.75 Ret sale cos & toltries spec strs | 23.14 Manufacture of glass fibres |
| 47.81 Ret sale; stlls & mrkt fd,bev,tob | 23.19 Man & proc oth glas, inc tech gla |
| 49.10 Passngr rail transport, interurba | 23.20 Manufacture of refractory product |
| 49.20 Freight rail transport | 23.32 Man bricks, tiles & constr prod |
| 49.31 Urban & sub passngr land transpor | 23.41 Man ceramic hhold & ornm artcls |
| 49.39 Other passngr land transprt n.e.c | 23.42 Manuf of ceramic sanitary fixture |
| 49.41 Freight transport by road | 23.51 Manufacture of cement |
| 49.50 Transport via pipeline | 23.52 Manufacture of lime and plaster |
| 50.10 Sea & coastal pass water transpor | 23.61 Man conc prod for constrcn purp |
| 50.20 Sea & coastal freight watr trnspr | 23.62 Man plaster prod for constrcn pur |
| 50.30 Inland passenger water transport | 23.63 Manufof ready-mixed concrete |
| 50.40 Inland freight water transport | 23.70 Cutting, shaping & finishing ston |
| 51.10 Passenger air transport | 23.99 Man othr non-met min prod n.e.c. |
| 51.21 Freight air transport | 24.10 Man basic iron, steel & ferro-all |
| 52.10 Warehousing and storage | 24.20 Man holow prof & rltd fit of stee |
| 52.21 Serv actv incidental to land tran | 24.31 Cold drawing of bars |
| 52.22 Serv actv incidental to wter tran | 24.33 Cold forming or folding |
| 52.23 Serv actv incidental to air trans | 24.42 Aluminium production |
| 52.24 Cargo handling | 24.43 Lead, zinc and tin production |
| 52.29 Other transportation supp acts | 24.44 Copper production |
| 53.10 Post actv under univesl serv obli | 24.45 Other non-ferrous metal productio |
| 53.20 Other postal and courier acts | 24.46 Processing of nuclear fuel |
| 58.13 Publishing of newspapers | 24.51 Casting of iron |
| 60.10 Radio broadcasting | 24.52 Casting of steel |
| 60.20 Tv programming & broadcasting act | 24.53 Casting of light metals |
| 61.10 Wired telecomtions activities | 24.54 Casting of othr non-ferrous metal |
| 61.20 Wireless telecomtions activities | 25.11 Man met structs & parts of struct |
| 61.30 Satellite telecoms activities | 25.12 Manu doors and windows of metal |
| 61.90 Other telecomtions activities | 25.21 Manu cent heating radiators & boi |
| 63.11 Data proc, hosting & related actv | 25.29 Man oth tnks, resvrs & cont of me |
| 63.12 Web portals | 25.30 Manu of steam gen, exc CH boilers |
| 63.91 News agency activities | 25.40 Manuf of weapons and ammunition |
| 63.99 Other info service acts n.e.c. | 25.50 Forg, press, stamp & roll-form me |
| 64.11 Central banking | 25.61 Treatment and coating of metals |
| 64.19 Other monetary intermediation | 25.62 Machining |
| 64.92 Other credit granting | 25.71 Manufacture of cutlery |
| 66.11 Administration of financial markt | 25.72 Manufacture of locks and hinges |
| 77.31 Rentng & leasng agr mchnry & eqmt | 25.73 Manufacture of tools |
| 81.21 General cleaning of buildings | 25.91 Man steel drums & sim containers |
| 81.22 Other building & ind cleaning act | 25.92 Manuf of light metal packaging |
| 81.29 Other cleaning activities | 25.93 Man of wire prods, chain & spring |
| 82.20 Activities of call centres | 25.94 Man of fasteners & screw mchn pro |
| 84.12 Reg of actv providing social serv | 25.99 Man other fabr metal prod n.e.c. |
| 84.21 Foreign affairs | 26.11 Manufof electronic components |
| 84.22 Defence activities | 26.12 Manuf of loaded electronic boards |
| 84.23 Justice and judicial activities | 26.20 Manuf computers & peripheral eqmt |
| 84.24 Public order and safety activitie | 26.30 Manuf of communication equipment |
| 84.25 Fire service activities | 26.40 Manuf of consumer electronics |
| 84.30 Complsry social security activs | 26.51 Man instr for meas, testing & nav |
| 85.10 Pre-primary education | 26.52 Manufacture of watches and clocks |
| 85.20 Primary education | 26.60 Man irradiation & electromed eqmt |
| 85.31 General secondary education | 26.70 Man opt instruments & photo eqmt |
| 85.32 Techl & vocational secondary educ | 26.80 Manu magnetic and optical media |
| 86.10 Hospital activities | 27.11 Manu of elect motors, gen & trans |
| 86.21 General medical practice activs | 27.12 Man elctrcty dist & cont apparatu |
| 86.22 Specialist medical practice activ | 27.20 Manu batteries and accumulators |
| 86.23 Dental practice activities | 27.31 Manufacture of fibre optic cables |
| 86.90 Other human health activities | 27.32 Man oth elctrnc & elec wirescable |
| 87.10 Residential nursing care activs | 27.33 Manufacture of wiring devices |
| 87.20 Res care activs for mental health | 27.40 Manu electric lighting equipment |
| 87.30 Res care actv for the eldly & dis | 27.51 Manu electric domestic appliances |
| 87.90 Other residential care activities | 27.52 Manu of non-electric domestic app |
| 88.10 Soc wrk act wo accm fr eld & disb | 27.90 Manu of other electrical eqmt |
| 88.91 Child day-care activities | 28.11 Man eng & turb, ex airvehcyc eng |
| 88.99 Other soc work actv wo accom nec | 28.12 Manuf of fluid power equipment |
| 94.91 Activs of religious organisations | 28.13 Man other pumps and compressors |
|  | 28.14 Manuf of other taps and valves |
|  | 28.15 Man bear, gear, grng & drvng elmn |
|  | 28.21 Man ovens, furnaces & furnace bur |
|  | 28.22 Manu lifting & handling equipment |
|  | 28.23 Man off mchn & eqmt exc PC & acc |
|  | 28.24 Manuf of power-driven hand tools |
|  | 28.25 Man non-dom cooling & ventiln eqm |
|  | 28.29 Man other gen-purp machinry n.e.c |
|  | 28.30 Man agricultural & forestry mchnr |
|  | 28.41 Manuf of metal forming machinery |
|  | 28.49 Manufacture of other machine tool |
|  | 28.91 Manuf of machinery for metallurgy |
|  | 28.92 Man mchnry for mng, quarr & const |
|  | 28.93 Man mcnry for food, bev & tob pro |
|  | 28.94 Man mchn for txt, app & lethr pro |
|  | 28.95 Man mchnry for pper & pperbrd pro |
|  | 28.96 Man plastics and rubber machinery |
|  | 28.99 Man othr spec-purp mchnry n.e.c. |
|  | 29.10 Manufacture of motor vehicles |
|  | 29.20 Man bodies for motor veh & traile |
|  | 29.31 Man of electric eqmt for motor ve |
|  | 29.32 Man othr parts & acc for motor ve |
|  | 30.11 Buildng of ships & floating struc |
|  | 30.12 Buildng pleasure & sportng boats |
|  | 30.20 Manu railway loco & rolling stock |
|  | 30.30 Manu air & spacecraft & rel mchnr |
|  | 30.40 Manuf military fighting vehicles |
|  | 30.91 Manufacture of motorcycles |
|  | 30.92 Manu bicycles & invalid carriages |
|  | 31.01 Manuf of office and shop furnitur |
|  | 31.02 Manufacture of kitchen furniture |
|  | 31.03 Manufacture of mattresses |
|  | 31.09 Manufacture of other furniture |
|  | 32.11 Striking of coins |
|  | 32.12 Manu jewellery & related articles |
|  | 32.13 Man imitation jewellery & rltd ar |
|  | 32.20 Manufacture of musical instrument |
|  | 32.30 Manufacture of sports goods |
|  | 32.40 Manufacture of games and toys |
|  | 32.99 Other manufacturing n.e.c. |
|  | 33.11 Repair of fabricated metal prodts |
|  | 33.12 Repair of machinery |
|  | 33.13 Repair of electrnc & optical eqmt |
|  | 33.14 Repair of electrical equipment |
|  | 33.15 Repair & maintenance ships & boat |
|  | 33.16 Repair & main aircraft & spacecrf |
|  | 33.17 Repair & main trnsport eqmt n.e.c |
|  | 33.19 Repair of other equipment |
|  | 33.20 Installation ind mchnry & equipmn |
|  | 35.30 Steam and air conditioning supply |
|  | 38.31 Dismantling of wrecks |
|  | 38.32 Recovery of sorted materials |
|  | 41.10 Development of building projects |
|  | 41.20 Constr of res and non-res buildng |
|  | 42.11 Construction of roads and motrway |
|  | 42.12 Constr railwys & undgrnd railwys |
|  | 42.13 Constructn of bridges and tunnels |
|  | 42.21 Constr of utility proj for fluids |
|  | 42.22 Constr util proj for elec & telco |
|  | 42.91 Construction of water projects |
|  | 42.99 Constr other civil eng proj n.e.c |
|  | 43.11 Demolition |
|  | 43.12 Site preparation |
|  | 43.13 Test drilling and boring |
|  | 43.21 Electrical installation |
|  | 43.22 Plumbng, heat & air-con installat |
|  | 43.29 Other construction installation |
|  | 43.31 Plastering |
|  | 43.32 Joinery installation |
|  | 43.33 Floor and wall covering |
|  | 43.34 Painting and glazing |
|  | 43.39 Othr buildng completn & finishing |
|  | 43.91 Roofing activities |
|  | 43.99 Othr specsd constr actv n.e.c. |
|  | 45.20 Maintenance & repair motor vehles |
|  | 45.31 Wsale trade motor veh parts & acc |
|  | 45.32 Ret trade of motor veh parts & ac |
|  | 45.40 Sale, main, rep mtrcycle & rel pr |
|  | 46.12 Agnts inv sale fuelmetind chem |
|  | 46.13 Agnts inv in sale timb & bldng ma |
|  | 46.14 Agnts inv sale ind eqmtshipsairc |
|  | 46.15 Agnts inv sale hhold gdsironmngry |
|  | 46.16 Agnts inv sale text & lether good |
|  | 46.18 Agnts specsd sale othr part prod |
|  | 46.19 Agents inv in sale variety goods |
|  | 46.21 Wsale grainuman tobseedsanml fd |
|  | 46.22 Wholesale of flowers and plants |
|  | 46.34 Wholesale of beverages |
|  | 46.39 Non-spec wsale of food, bev & tob |
|  | 46.41 Wholesale of textiles |
|  | 46.42 Wholesale of clothing and footwea |
|  | 46.43 Wsale of electrical household app |
|  | 46.44 Wsale china & glasswre & clean ma |
|  | 46.45 Wholesale of perfume and cosmetic |
|  | 46.47 Wsale furn, carpts & lightng eqmt |
|  | 46.48 Wholesale of watches and jeweller |
|  | 46.49 Wholesale of other household good |
|  | 46.51 Wsale comp, comp perp eqmt & sftw |
|  | 46.52 Wsale elctrnc & telecom eqmt & pr |
|  | 46.61 Wsale of agric mchnry, eqmt & sup |
|  | 46.62 Wholesale of machine tools |
|  | 46.63 Wsale mining, cons & civ eng mcnr |
|  | 46.64 Wsale of mchnry for textile ind |
|  | 46.65 Wholesale of office furniture |
|  | 46.66 Wsale of other off machinry & eqm |
|  | 46.69 Wsale of other machinery & eqmt |
|  | 46.71 Wsale solliqgas fuel & rltd prod |
|  | 46.72 Wholesale of metals and metal ore |
|  | 46.73 Wsale wood, constr mat & san eqmt |
|  | 46.74 Wholesale of DIY eqmt & supp |
|  | 46.75 Wholesale of chemical products |
|  | 46.76 Wsale of other intermediate prod |
|  | 46.77 Wholesale of waste and scrap |
|  | 46.90 Non-specialised wholesale trade |
|  | 47.19 Oth ret sale in non-spec stores |
|  | 47.25 Ret sale of bev in spec stores |
|  | 47.26 Ret sale of tob prod in spec stre |
|  | 47.30 Ret sale of auto fuel in spec str |
|  | 47.52 Ret sale hardware eqmt spec strs |
|  | 47.62 Ret sale newsp & stat in spec str |
|  | 49.32 Taxi operation |
|  | 49.42 Removal services |
|  | 58.14 Publishng of journls & periodicls |
|  | 62.03 Computer facilities mangmnt actv |
|  | 64.30 Trusts, funds & sim financial ent |
|  | 66.21 Risk and damage evaluation |
|  | 68.32 Mgmt real estate on feecont basis |
|  | 71.20 Technical testing and analysis |
|  | 72.11 Res & experimental dev on biotech |
|  | 72.19 Othr R&D on natural sciences & en |
|  | 75.00 Veterinary activities |
|  | 77.11 Rent & lease cars & light motr ve |
|  | 77.12 Renting and leasing of trucks |
|  | 77.21 Rentng & leasing rec & sport good |
|  | 77.29 Rent & lease othr per & hhold goo |
|  | 77.32 Rentleas constr & eng mchn & eqmt |
|  | 77.33 Rentlease off mchn & eqmt inc PC |
|  | 77.34 Rentng & leasng watr trnprt eqmt |
|  | 77.35 Rentng & leasng air transprt eqmt |
|  | 77.39 Rentleas mchn,eqmt & tang gds nec |
|  | 80.10 Private security activities |
|  | 80.20 Security systems service activs |
|  | 80.30 Investigation activities |
|  | 81.10 Combined facils support activitie |
|  | 81.30 Landscape service activities |
|  | 82.19 Copyng,doc prep & othr off sup ac |
|  | 82.92 Packaging activities |
|  | 92.00 Gambling and betting activities |
|  | 96.01 Wash & (dry)cleang text & fur pro |
|  | 96.03 Funeral and related activities |
| **Group III: none-key workers - shut down business** | **Group IV: none-key workers: home office** |
| 45.11 Sale of cars & light motor vehles | 58.11 Book publishing |
| 45.19 Sale of other motor vehicles | 58.12 Publ of directs & mailing lists |
| 47.41 Ret sale PC eqmt & acc spec strs | 58.19 Other publishing activities |
| 47.42 Ret sale of telcom eqp spec store | 58.21 Publishing of computer games |
| 47.43 Ret sale aud & vid eqmt spec strs | 58.29 Other software publishing |
| 47.51 Ret sale of text in specsd stores | 59.11 Motn pic, vid & tv prog prod actv |
| 47.53 Ret sale of d?r eqmt spec stores | 59.12 Motn pic, vid & tv prog po-pro ac |
| 47.54 Ret sale of white goods spec stre | 59.13 Motn pic, vid & tv prog dist actv |
| 47.59 Ret sale of fixfit in spec strs | 59.20 Sound recording & music publ actv |
| 47.61 Ret sale of books in specsd store | 62.01 Computer programming activities |
| 47.63 Ret sale mus & vid rec spec strs | 62.02 Computer consultancy activities |
| 47.64 Ret sale of sprt eqmt in spec str | 62.09 Other IT & computer service actv |
| 47.65 Ret sale of games & toys spec str | 64.20 Activities of holding companies |
| 47.71 Ret sale of clothing in spec stre | 64.91 Financial leasing |
| 47.72 Ret sale ftwr & lthr gds spec str | 64.99 Oth fin ser,exc ins & pen fund,ne |
| 47.76 Ret sale flwrs & pets in spec str | 65.11 Life insurance |
| 47.77 Ret sale jewlry items in spec str | 65.12 Non-life insurance |
| 47.78 Oth ret sale new gds in spec strs | 65.20 Reinsurance |
| 47.79 Ret sale of secnd-hnd goods in st | 65.30 Pension funding |
| 47.82 Ret sale; stlls & mrkt clthg, ftw | 66.12 Sec & commodity contrcts brokerag |
| 47.89 Ret sale via stalls & mrkt oth gd | 66.19 Oth act ax fin ser,ex in & pen fn |
| 47.91 Ret sale mail order houses, intrn | 66.22 Actv of insurance agents & broker |
| 47.99 Othr ret sale exc stores etc | 66.29 Othr actv aux to ins & pensn fndn |
| 55.10 Hotels and similar accommodation | 66.30 Fund management activities |
| 55.20 Holiday and other short stay acco | 68.10 Buying and selling own real estat |
| 55.30 Cmpg grnd, rec veh prk & trail pr | 68.20 Renting & op ownleasd real estate |
| 55.90 Other accommodation | 68.31 Real estate agencies |
| 56.10 Restnt & mobile food servc actv | 69.10 Legal activities |
| 56.21 Event catering activities | 69.20 Accntng & auditng actv;tax consul |
| 56.29 Other food service activities | 70.10 Activities of head offices |
| 56.30 Beverage serving activities | 70.21 PR & communication activities |
| 59.14 Motn picture projection activitie | 70.22 Bus & other mangmnt constny actv |
| 82.30 Convntn and trade show organisers | 71.11 Architectural activities |
| 85.51 Sports and recreation education | 71.12 Eng actv & related tech consltncy |
| 85.52 Cultural education | 72.20 R&D on social sci and humanities |
| 85.53 Driving school activities | 73.11 Advertising agencies |
| 85.59 Other education n.e.c. | 73.12 Media representation |
| 85.60 Educational support activities | 73.20 Market resch & pub opinion pollin |
| 90.01 Performing arts | 74.10 Specialised design activities |
| 90.02 Supprt activs to performing arts | 74.20 Photographic activities |
| 90.03 Artistic creation | 74.30 Transltion and interpretation act |
| 90.04 Operation of arts facilities | 74.90 Otr prof,scntfc & tech actv n.e.c |
| 91.01 Library and archive activities | 77.40 Lease intel prop, exc cpyrghtd wr |
| 91.02 Museum activities | 78.10 Actv of emplymnt placment agncies |
| 91.03 Op of hist sites & sim vis atrctn | 78.20 Temp employment agency activities |
| 91.04 Bot & zoolgicl grdns & nat res ac | 78.30 Other human resources provision |
| 93.11 Operation of sports facilities | 79.11 Travel agency activities |
| 93.12 Activities of sport clubs | 79.12 Tour operator activities |
| 93.13 Fitness facilities | 79.90 Othr reservtn serv & related actv |
| 93.19 Other sports activities | 82.11 Combined office admin service act |
| 93.21 Act of amusement park & theme par | 82.91 Actv coll agncies & credit bureau |
| 93.29 Other amusement and rec activitie | 82.99 Other bus supp service actv n.e.c |
| 94.11 Act of busness & employrs memb or | 84.11 General public admin activities |
| 94.12 Activities of prof mem org | 84.13 Reg & contr to mre eff op of busi |
| 94.20 Activities of trade unions | 85.41 Post-secndry non-tertiary educatn |
| 94.92 Activs of political organisations | 85.42 Tertiary education |
| 94.99 Activities of other mem org n.e.c | 97.00 Act hhold as emplyers of dom pers |
| 95.11 Repair of comps & peripheral eqmt | 98.10 Undif good-prod act of priv hhold |
| 95.12 Repair of communication equipment | 98.20 Undif serv-prod act of priv hhold |
| 95.21 Repair of consumer electronics |  |
| 95.22 Rep hhold apps & home & grden eqm |  |
| 95.23 Repair of footwr and leather good |  |
| 95.24 Rep of furnitre & home furnishngs |  |
| 95.25 Rep of watches, clocks & jeweller |  |
| 95.29 Rep of other personl & hhold good |  |
| 96.02 Hairdressng & othr beauty treatmn |  |
| 96.04 Physical well-being activities |  |
| 96.09 Other personal service actv n.e.c |  |
| Note: Numbers refer to four digit code of main industry class code in the ONS Labour Force Survey (20). | |
